# Intrinsic Influences on Medical Emergency Team Call Stand‐Down Decision‐Making: An Observational Study

**DOI:** 10.1111/jan.70148

**Published:** 2025-09-16

**Authors:** Natalie A. Kondos, Jo McDonall, Jonathan Barrett, Tracey Bucknall

**Affiliations:** ^1^ School of Nursing and Midwifery, Faculty of Health Deakin University Geelong Australia; ^2^ Centre for Quality and Patient Safety Research, Institute for Health Transformation Deakin University Geelong Australia

**Keywords:** clinical decision‐making, critical care outreach, ICU liaison nurse, intrinsic influences, medical emergency team (MET), nursing, rapid response systems (RRS)

## Abstract

**Aim:**

The aim of this research was to describe factors that influence Intensive Care Unit liaison nurses' decision to stand down a medical emergency team call response. The decision to end a medical emergency team response for a deteriorating patient is referred to as the medical emergency team call stand‐down decision. Intensive Care Unit liaison nurses, also known internationally as critical care outreach nurses, make medical emergency team call stand‐down decisions in complex and challenging clinical environments. However, the factors influencing these decisions are not well described in the literature.

**Design:**

Exploratory descriptive qualitative study.

**Methods:**

Seven Intensive Care Unit liaison nurses who attended medical emergency team calls in a large acute metropolitan tertiary referral public hospital, with a mature three‐tiered rapid response system, were observed and interviewed. Observations of 50 medical emergency team call responses and 50 post medical emergency team call interviews were conducted between March 2022 and August 2022. Findings were analysed using inductive content analysis.

**Results:**

Intensive Care Unit liaison nurse decisions to stand down MET call responses were influenced by three intrinsic factors: (1) propositional knowledge, (2) experiential knowledge, (3) situational knowledge and information processing styles. Intensive Care Unit liaison nurses utilised these intrinsic factors to support their decision to terminate medical emergency team call response.

**Conclusion:**

This study explored the intrinsic influences on individual Intensive Care Unit liaison nurses in deciding to end a medical emergency team call. By highlighting these individual influences on decision‐making, the findings may be used to support medical emergency team responders educational needs and identification of potential heuristics and biases inherent in clinical decision‐making which contribute to adverse events.

**Patient or Public Contribution:**

No patient or public contribution.

**Implications for Profession and/or Patient Care:**

By understanding the influences on an individual's clinical decision‐making, strategies can be put in place for educational development and support for experiential learning. The study highlights areas of potential bias and heuristic use that may lead to sub‐optimal clinical decisions and increased risk for deteriorating patients. Research findings can be applied internationally to a range of rapid response systems and critical care outreach teams that respond to deteriorating patients.

**Reporting Method:**

The consolidated criteria for reporting qualitative research (COREQ) guidelines were used for reporting this study.

**Summary**

- What is known?
  ○ Many factors influence nurses' decision‐making in the clinical context
  ○ Patients who have repeated episodes of deterioration in acute settings have worse outcomes than those who have a single episode of deterioration.
  ○ Decision‐making is influenced by many individual factors, including the use of different knowledge types and distinct informational processing styles.
- What this paper adds?
  ○ This study showed that propositional, experiential, and situational knowledge are three intrinsic factors that influence Intensive Care Unit liaison nurses' decisions to stand down a medical emergency team call.
  ○ Highlights that individual information processing styles influence intensive care unit liaison nurses' decision to end a medical emergency team call.
  ○ Enhances awareness regarding current decision‐making practices and may lead to strategies that will improve clinicians' decisions and ultimately improve patient care.
- What does this paper contribute to the wider global clinical community?
  ○ Description of the intrinsic influences on Intensive Care Unit liaison nurses' decision‐making process to de‐escalate care and end medical emergency team call responses.
  ○ The optimisation of medical emergency team call stand‐down decision‐making practice requires a strategy that develops an individual's decision‐making during dynamic clinical environments to ensure a positive impact on patient outcomes.

## 1 Introduction

Decision‐making involves assessing and analysing relevant information to arrive at a conclusion or judgement about future behaviour (Bucknall et al. 2007). Clinical decision‐making involves identifying, comparing, and assessing relevant information to develop an informed opinion or decision regarding a patient's future treatment or care plans (Hutchinson and Bucknall 2016). Medical emergency team (MET) clinicians' make decisions to end a MET call response for a deteriorating patient once there is a reduction in patient risk or when another concurrent MET call is activated (Cheung et al. 2019; DeVita et al. 2006). Factors that influence the individual ICU liaison nurse's decision to end a MET call response have not been described in the literature. The literature highlights decision‐making influences as being either intrinsic to an individual nurse's decision‐making or extrinsic; that is, the external influences on nurses decision‐making in the environment, such as staffing, workloads, relationships (Bucknall et al. 2007). Both intrinsic and extrinsic influences were captured in the research; however, for reporting clarity, this article only reports the intrinsic influences on ICU liaison nurses stand‐down decision‐making. Extrinsic influences will be reported in another article to allow for in‐depth analyses on each category of influence.

## 2 Background

The term Medical Emergency Team (MET) refers to the team that responds to MET calls and Code Blue (cardiac arrest) calls (Jones 2023; Jones et al. 2023). MET is the most popular model of Rapid Response Team (RRT) used in Australia (DeVita et al. 2006, 2010; Jones 2023). The MET is usually comprised of medical and nursing staff from critical care backgrounds whose specific role is to respond to and manage patient deterioration (Barbetti and Lee 2008; Hourihan et al. 1995; Lee et al. 1995, 2021; Silva et al. 2016).

Many MET services in Australian major metropolitan health services are ICU liaison nurse led (Eliott et al. 2012, 2008). The role of the ICU liaison nurse may include the following activities: responding to MET calls and code blue activations, advising on and managing deteriorating patients in wards, supporting staff with patients recently discharged from ICU, and formal and informal education and skills training for general ward staff (Eliott et al. 2012, 2008; Endacott et al. 2009; Sjöstedt et al. 2022).

For over ten years, more than 95% of Australian hospitals with an Intensive Care Unit (ICU) have implemented an RRT, resulting in upwards of 92,000 RRT evaluations (Orosz et al. 2020). In Australia, research evidence suggests more than 25% of patients admitted to hospitals experience acute deterioration that necessitates urgent clinical review and frequently intervention from a rapid response team (RRT), such as a Medical Emergency Team (MET) (Bucknall et al. 2022). A substantial retrospective study found that the frequency of MET calls was 89 MET activations for every 1000 admissions (Levkovich et al. 2019).

A MET response may result in three different clinical outcomes: 1) the precipitating cause may be resolved, resulting in no further need for escalation (Still et al. 2018) 2) unresolved deterioration, with the patient being transferred to a higher level of care or for treatment; or 3) patients have intermittent episodes of deterioration requiring repeat MET call escalation (Chalwin et al. 2019; Na et al. 2020). Further patient deterioration may be a result of patient factors such as a persistent and complex pattern of decline (Na et al. 2020; Stelfox et al. 2014), anthropogenic factors, such as alarm and escalation fatigue, communication issues, biassed clinician observation(s), or organisational factors related to system constraints (Berwick et al. 2006; Runciman et al. 2012). Patients whose condition continues to deteriorate and require repeated MET calls have poorer outcomes, such as an increased hospital length of stay (LoS), increased incidence of transfer to a higher level of care, and higher mortality rates (Chalwin et al. 2019; Na et al. 2020; Stelfox et al. 2014). Given the adverse consequences of repeated deterioration, this study sought to identify the intrinsic factors that influence the intensive care unit (ICU) liaison nurses' decision to stand‐down a MET response. Understanding the intrinsic influences on nurses' decision‐making may assist in the development of strategies to optimise MET call stand‐down decisions and potentially reduce the incidence of further clinical deterioration. This in turn will improve patient outcomes and improve the capacity of MET to respond to other patients experiencing clinical deterioration.

## 3 The Study

### 3.1 Aim

The aim was to examine the intrinsic factors that influence ICU liaison nurses' decision‐making process for MET call stand‐down.

## 4 Methods

### 4.1 Design

Naturalistic observations with follow‐up interviews were undertaken to explore the previously undescribed MET call stand‐down phenomenon and the meanings that the ICU liaison nurse participants place on the MET call stand‐down decision (Jorgensen 1989, 2015). To support the triangulation of data and minimise the influence of the non‐participant observer on data collection (Cole 2005), post‐MET call observation interviews were conducted. This provided an opportunity for the corroboration and contextualisation of data collected during observations by those who were being observed (Graneheim et al. 2017).

The theoretical underpinning of this study was decision‐making theory. Decision‐making is a process where individuals, groups, or organisations choose from available alternatives by gathering and evaluating relevant information (Carroll and Johnson 1990). It involves various mental activities that help recognise decision scenarios and weigh preferences to produce judgements (Einhorn and Hogarth 1981; Kahneman and Tversky 1979). The MET call stand‐down decision encompasses multiple levels of decision‐making, primarily focused on whether to end a MET call. Consideration must be given to the consequences of making this choice without a comprehensive understanding of the patient's situation, established management and escalation plans, or the risks of acting prematurely, which could lead to further patient deterioration. The consolidated criteria for reporting qualitative research (COREQ) guidelines were used to report this study (File S1) (Tong et al. 2007).

### 4.2 Setting

The study took place at one metropolitan acute tertiary referral public hospital, with a mature three‐tiered rapid response system. The hospital has approximately 650 acute admission beds and over 9000 MET calls annually.

### 4.3 Recruitment

During the data collection period, all seven ICU liaison nurses working at the hospital were recruited. The primary function of an ICU liaison nurse role is to respond to acute patient deterioration following a MET call and Code Blue activation. Their secondary role is to support ward staff caring for deteriorating patients requiring escalation in treatment and the follow‐up of patients recently discharged from the ICU.

Following ethical approval, the hospital's ICU medical and nursing clinical leads sent an email to the ICU liaison nurses and invited them to participate. The email contained a brief study overview and a Plain Language Statement (PLS), outlining the study aims and expectations of participating in the study. Participants were notified that the researcher would observe random MET calls and conduct semi‐structured interviews at the conclusion of the MET call or soon after if another organisational need arose. Study participants were informed that they could opt out at any time; no participants chose to opt out of any of the observations or interviews conducted. Patients and other staff attending the MET call had consent waived by the ethics committee as they were not the focus of the study.

### 4.4 Exclusion Criteria

- MET responses where patients were documented for terminal care.
- Patients isolated under airborne precautions to minimise the potential transmission risk for COVID‐19.

### 4.5 Instruments

Running commentaries were audio‐recorded in real‐time on a digital voice recorder. This involved the student researcher vocalising what they observed in terms of the actions, communication, MET call stand‐down decision, and decision‐making practices of the ICU liaison nurse participating in the MET call. An opening question was used to guide the interviews to assist in exploring the factors that influence the ICU liaison nurse's decision to stand‐down the MET response. ‘When you decided to stand‐down the MET call, what influenced your decision to end it?’ Then depending on what had occurred in the observation, the interviewer explored the influences and used prompts such as ‘can you tell me more about that?’ The recordings were transcribed verbatim after the MET call observations and interviews. Field notes made after the observations and interviews also detailed the student researcher's reflective information, that is, thoughts, ideas, questions, and issues during the observations and interviews.

### 4.6 Data Collection

#### 4.6.1 Procedure

The aim was to observe a total of 50 MET call responses across the MET service hours (0800–2400) over a five‐month period. This number of observations ensured a stratified sample was used to explore differences between patients experiencing clinical deterioration and ICU nurse clinician participants making these stand‐down decisions. Observations and interviews were conducted from 0800 to 2400 h (MET service hours) and spread evenly across all weekdays (Monday through to Sunday). Two separate observers undertook five pilot observations prior to study commencement to enhance observational and interview data validity via interrater reliability testing (Armstrong et al. 1997).

All MET calls have'real‐tim' notifications relayed via the hospital's overhead speaker alert system. The MET response team is comprised of one ICU liaison nurse and sometimes an ICU registrar if called upon, with the response activated by calling a dedicated phone line for all hospital emergencies. The student researcher was present in the hospital on scheduled days and attended each call when the notification was announced. The student researcher assumed the role of non‐participant observer to reduce bias within the setting of the MET call.

**FIGURE 1:**
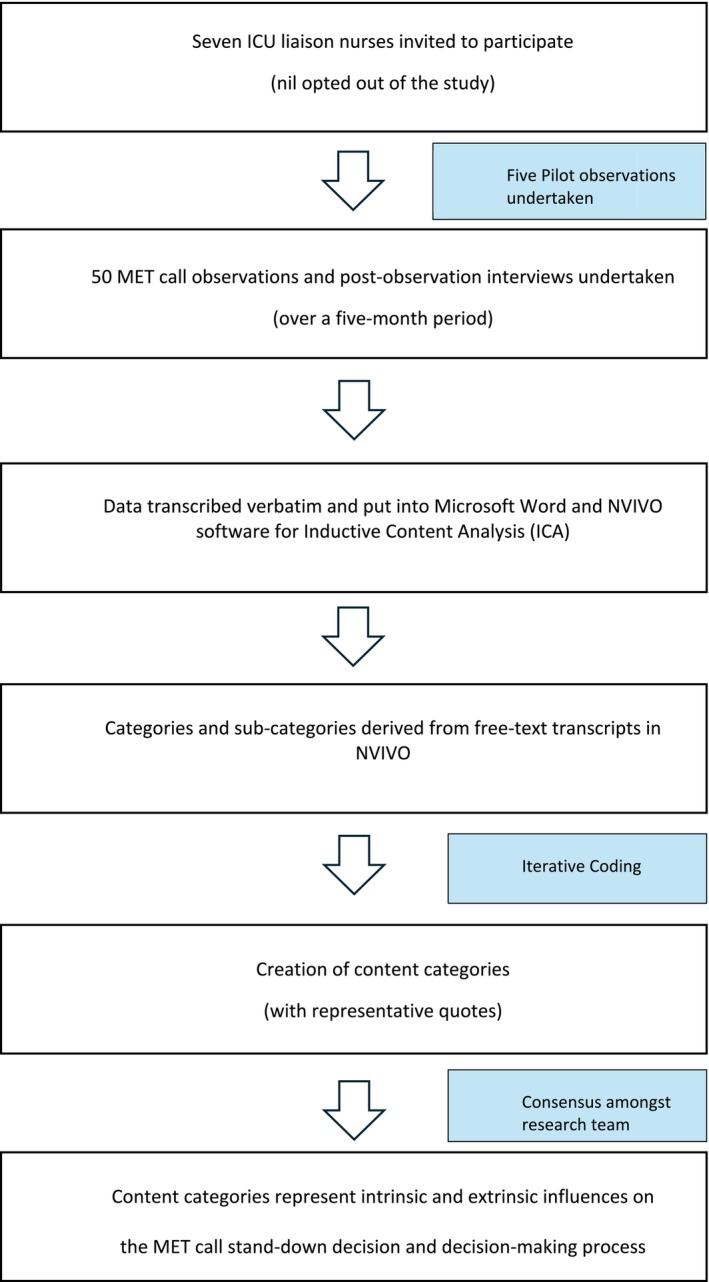
Data collection and analysis process for MET call observations and interviews.

Post‐MET call interviews were conducted with each ICU liaison nurse immediately after ending the MET response to enable prompt description and follow‐up information from the individuals being observed (Creswell 2013). No repeat interviews were necessary; phone call interruptions were managed within the allocated interview time (up to 15 min), and interviews were recommenced as appropriate. The interviews were audio‐recorded with permission from the ICU liaison nurse participants. Figure 1 outlines the data collection procedure for this study.

### 4.7 Data Analysis

All data were deidentified, with each participant allocated a unique code to maintain privacy and confidentiality. A second researcher undertook interrater reliability testing for the first five MET call observations and interviews to ensure that the data collected was descriptive and reproducible and that the interview proforma generated the same responses from the participants.

The audio recordings were transcribed verbatim and converted into NVIVO (version 11) data files for review and analysis. Microsoft Excel (version 360) and SPSS software (version 27.0) were used for demographic data analysis. Data analysis aimed to create a comprehensive summary and understanding of the content from observations and interviews in each dataset (Bennett et al. 2019; Vears and Gillam 2022).

Inductive content analysis (ICA) was used to analyse the qualitative data by identifying patterns, themes, and categories (Vears and Gillam 2022). ICA allows for the development of new categories and concepts from the data itself (Bennett et al. 2019; Vears and Gillam 2022). This approach is often used when the research topic is exploratory or when there is limited existing literature on the topic (Vaismoradi et al. 2013) as was the case in this study. This method was selected as little is known about the decision‐making practice, process, and influences on the decision to end a MET call response (Elo and Kyngäs 2008). Each of the MET observations and linked interviews were analysed as a unit for all 50 encounters.

**FIGURE 2:**
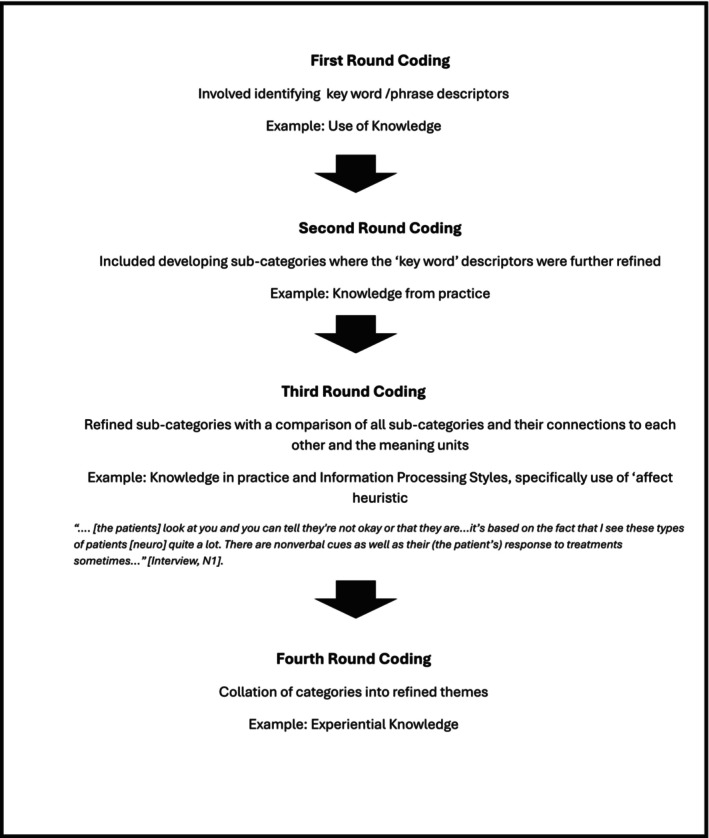
A flow chart illustrating the process of inductive content analysis used to analyse observation and interview data.

Categories and sub‐categories were derived from the data without predetermined themes. The process included reading and annotating the transcripts, segmenting the data, and analysing the segments through coding, comparison, and grouping (Vears and Gillam 2022). Content categories were established through iterative coding and consensus amongst the research team to minimise bias. Coding was approached in a step‐wise process: Step 1: reading and understanding transcript content to support comprehension; Step 2: first round coding involved identifying ‘bigger picture’ meaning units or a class or type of broad category content (chunks of text) that were relevant to the research questions; Step 3: second round coding included developing sub‐categories where the ‘bigger picture’ categories were broken up into line‐by‐line coding; Step 4: refining fine‐grained sub‐categories of the ‘bigger picture’ categories and a comparison of all sub‐categories and their connections to each other and the bigger picture meaning units; Step 5: synthesis and interpretation which included connecting categories to support an overall explanation and description of the phenomenon and a synthesis of all content categories (Vears and Gillam 2022). Representative quotes were selected for each category and sub‐category related to the influences on the decision to end a MET call (Vears and Gillam 2022). Figure 1 provides the data analysis procedure for this study and Figure 2 outlines the process of inductive content analysis.

### 4.8 Rigour and Reflexivity

The study's methodological rigour was upheld by meeting the confirmability criteria, which encompassed factors like auditability, credibility, fittingness, and applicability, widely acknowledged in qualitative research (Sandelowski 1986; Schwandt et al. 2007). Auditability was ensured by establishing predetermined study objectives, using a third party to select participants (Vears and Gillam 2022), employing unique case numbers to de‐identify data (Elo and Kyngäs 2008; Vears and Gillam 2022), and transcribing, managing, and analysing data using NVIVO Pro software. The research team also took measures to ensure representativeness of data, utilised triangulation, and cross‐checked descriptions to encompass both typical and atypical aspects of the phenomenon to address credibility and fittingness, specifically addressing the risks of holistic fallacy, elite bias, and ‘going native’ (Sandelowski 1986).

The student researcher received training in data collection and qualitative methods and conducted all observations and interviews. As the student researcher had a professional relationship with the participants, efforts were made to mitigate potential influence by informing participants of the researcher's purpose and study aim. Furthermore, risks to participants, the student researcher's background and potential biases, were disclosed through an introductory email and Plain Language Statement (PLS). A reflexive diary was also kept by the student researcher and discussed intermittently with the research team, with these conversations addressing assumptions and biases. Collaboration with the research team and reflexive writing were used by the student researcher to enhance the awareness of the researcher's own perspectives and assumptions regarding the research process, identify critical interpersonal dynamics impacting the participants and their data, and to record, probe and build on analytical insights (Olmos‐Vega et al. 2023).

### 4.9 Ethical Considerations

Human Research Ethics Committee approval was obtained from the Hospital (No. 26/20) and was noted by the university's Human Ethics Advisory Group (HEAG; ref. 2025/HE00545). The project was deemed low risk. Patient safety was an important consideration during the MET call observations. If the researcher felt that the observation impacted patient safety at any time, the observation would be abandoned, with clear documentation of this. No observations were abandoned for this study.

## 5 Findings

### 5.1 Participant Characteristics

Seven ICU liaison nurses were observed attending a total of 50 MET responses; each nurse was interviewed at the conclusion of each MET response. The demographic characteristics of the nurse participants are presented in Table 1. The interviews ranged from 8 to 15 min, with an average duration of 13.6 min. Most ICU liaison nurses were female (*n* = 6, 86%), aged between 41 and 50 years old, held a critical care graduate certificate or higher, and had more than 16 years' experience in the role.

**TABLE 1:** Demographic characteristics of participants (*N* = 7).

Participants (n)	*n* (%)
Characteristic	
Sex	
Female	6 (86)
Male	1 (14)
Age	
31–40 years	1 (14)
41–50 years	5 (70)
51–60 years	1 (14)
Experience working as ICU liaison Nurse	
5–10 years	3 (43)
11–15 years	0 (0)
16–20 years	4 (57)
Highest level of education	
Postgraduate certificate (critical care)	4 (57)
Postgraduate diploma (critical care)	1 (14)
Masters (critical care)	2 (29)

### 5.2 MET Response—Patient Characteristics

The number and type of MET responses (single/repeat) are presented in Table 2. There were 19 (38%) patients who required a single MET call and 31 (62%) patients who required repeated MET calls (i.e., ≥ 2 MET calls). Each ICU liaison nurse attended both single and repeat MET calls during the data collection period. It was recorded in field notes if another MET call was activated during the initial MET call being observed and noted as a ‘concurrent MET call’. The concurrent MET calls were attended by the same ICU liaison nurse, as only one ICU liaison nurse covers the MET call service per shift. Even though there were differences in percentages between the two MET call patient groups, the frequency of concurrent MET calls activated was consistent across the single and repeat MET call patient groups.

**TABLE 2:** ICU liaison nurse attendance at MET calls and concurrent MET calls for single and repeat MET call patient observations (*N* = 50).

Observations (*N* = 50)	Single MET call	Repeat MET call
	*n* (%) 19 (100)	*n* (%) 31 (100)
ICU liaison nurse number & attendance		
N1	1 (5)	4 (13)
N2	5 (26)	6 (19)
N3	2 (11)	6 (19)
N4	1 (5)	5 (16)
N5	5 (26)	5 (16)
N6	2 (11)	3 (10)
N7	3 (16)	2 (7)
Concurrent MET call activated during MET call		
Yes	9 (47)	9 (29)
No	10 (53)	22 (71)

*Note:* Concurrent MET calls are ≥ 2 MET calls escalated simultaneously.

### 5.3 Intrinsic Influences on MET Call Stand‐Down Decision‐Making

The inductive analysis identified three categories of intrinsic knowledge that influenced the nurses' decision to stand‐down the MET response: 1. Propositional knowledge; 2. Experiential knowledge; 3. Situational knowledge; and 4. Information processing style. The content analysis method is presented in Figure 2.

#### 5.3.1 Propositional Knowledge

Propositional knowledge is defined as specialised critical care knowledge that ICU liaison nurses have obtained through postgraduate education which can be synthesised and applied in a time‐critical way. The ICU liaison nurses incorporated information from various sources including the patient, assessment findings, monitoring devices, hospital protocols, policies, and clinical practice guidelines to influence their decisions to end the MET response. When using propositional knowledge, participants reported considering the patient's clinical condition and dependence on treatment prior to making the decision to stand‐down, for example:

…leave the oxygen flow at fifteen litres [N1]… and ‘[the patient's] arterial oxygen level is very low, even though he is on 15 litres via the non‐rebreather mask’. [Observation N1]

In addition, participants reported knowledge of medication administration practices was an important consideration before standing down a MET, for example:

N4 …you can give the digoxin as an infusion rather than as a push (bolus)…. [Observation N4]

N4 is aware that administering digoxin as an infusion is generally safer for the patient than as a bolus, especially given the patient's blood pressure status. N4 understands that the digoxin order is for a loading dose that should be administered over a specific duration (10 min). This reflects knowledge about dosage regimens and administration times for this medication. N4 recognises the need to monitor the patient's blood pressure due to its current state, indicating an awareness of how digoxin administration can impact hemodynamic stability.

During the interviews, the nurses reflected that their decision to end the MET call was based on their propositional knowledge and their understanding of key patient assessment data, risk, and interventions required:

…his [the patient's] airway, breathing, circulation was all safe […]they [the doctors] were getting a plan to check his haemoglobin (Hb) because his [the patient's] blood pressure was low, so I was happy about that. There was some concern about his [the patient's] neurological state, but it seemed like he [the patient] was doing all the right things. [Interview, N5]

…the main thing I needed to see completed before I left, particularly for that patient was goals of care. Given that she [the patient] was clearly very unwell …and was really reaching the ceiling of her care on the ward. [Interview, N7]

Another nurse was observed to state Digoxin [cardioactive rate control medication] had been administered and the patient was responding as expected, therefore she could safely end the MET response:

[…]the MET call was initially called for tachycardia …the heart rate was 100‐110 bpm (beats per minute) when we first got there…his [the patient] heart rate had then dropped so he [the patient] was responding to the medications we gave him [Digoxin], it was for decreased heart rate that the MET call was ended. [Interview with N2]

From these examples it was observed that ICU liaison nurses used propositional knowledge to support their understanding of abnormal patient assessment findings and implementation of associated interventions. The initiation or completion of these interventions was observed to signal the beginning of the stand‐down decision‐making process with respect to ending the MET call response. This was also reflected in the post‐MET call interviews with the ICU liaison nurses, who indicated they used this knowledge when considering the decision to end the MET response.

#### 5.3.2 Experiential Knowledge

Experiential knowledge is described as knowledge obtained through repeated exposure to similar patient types/groups, MET diagnoses/syndromes, MET call trajectories, and management of patients. Experiential knowledge is used to support the decision to end a MET call based on pattern recognition and is gained through hands‐on experience with various patient types, diagnoses, and medical emergency scenarios. This type of knowledge is developed through repeated exposure and involvement in similar situations, allowing healthcare professionals to recognise patterns and make informed decisions based on past encounters.

Observed examples were:

N6 is explaining that this MET call was for an unwitnessed patient fall however it was very high risk due to the patients' medical condition, N6 states: ‘… I know that these types patients have a tendency to have clotting issues and platelet problems and therefore a higher risk of bleeding after a fall, so they [the bedside nurses] will need to get a haemoglobin (Hb), to get a better idea of what is happening with the patient before the decision is made to finish the MET call [N6]’. [Observation N6]

[N7] is informing the treating team resident that following assessment it is noted the patient has slightly unequal and sluggish right pupil and is reporting a frontal headache N7 states: ‘in my previous experience this could mean something serious is happening neurologically’ [N7]. She [N7] is getting a pen torch for the resident to assess the patient's pupillary response together. N7 has noted the patient has a cataract in their right eye, and this may provide an explanation for unequal pupils. As a result of this finding, N7 states: ‘[the patient] should be okay now’. [Observation N7]

The ICU liaison nurse participants further reflected about their use of experiential knowledge to support their decision to end MET calls in the post‐MET call follow‐up interviews:

…. [the patient] looks at you and you can tell they're not okay, […], it's based on the fact that I see these types of patients [neurology patients] quite a lot. There are nonverbal cues as well as their (the patient's) response to treatments sometimes. I've had a lot of experience with these types of patients [neuro patients] in ICU so I know when to end the MET call based on that. [Interview with N1]

Nurse participant [N2] explains she is familiar with neurology patients and relies on her experience and pattern recognition to assess the patient and their response to treatments. This expertise aids her in deciding when to conclude the MET call response.

A further example was ICU liaison nurse N2 who used her prior experience with patients with chronic obstructive pulmonary disease (COPD) to help her assess the patient's stability and the response to treatments at the tail end of the MET call. N2 was observed discussing her assessment with the bedside nurse and putting in place an intervention. N2 then waited to see a positive response in the patient's oxygen saturations before making the decision to end the MET call and leave the patient.

I probably should have left at that time, …, I would have left earlier usually but the resident [junior doctor] asked why he [the patient] was on high flow oxygen, and I could see that he [the patient] was quite a brittle COPD (chronic obstructive Pulmonary Disorder) patient and CO2 (carbon dioxide) retainer […] I see a lot of these patients in MET calls[…]and then I waited till his oxygen saturations were normal and then I left…. [Interview with N2]

#### 5.3.3 Situational Knowledge and Awareness

Situational knowledge evolves from repeated exposure to the same clinical areas and patients, which builds familiarity with people and an understanding and awareness of the environment. This practice of increasing their situational awareness and using this knowledge to make the decision to end a MET call was best represented by the following two observations:

In this observed scenario N5 was engaged in a discussion with senior doctors regarding the treatment plan for a patient experiencing both tachycardia and bradycardia that was based on previous experience of attending MET responses for this patient. The medical staff in the room [ICU registrar, treating team registrar and medical registrar] had abandoned the idea of giving amiodarone to manage the patient's tachycardia and have confirmed with N5 to give an IV (intravenous) bag of magnesium to replace electrolytes instead. *[Observation N5]; A*nd N2 addresses a critical issue relating to patient care in a specific inpatient area that is physically separated from the main hospital building, and it is a common occurrence that patients from this area frequently deteriorate requiring MET calls…N2 states ‘*they have tendency to fall under the radar of the treating medical team and that in their experience they often get missed’ [N2]* [Observation N2]

In both observations above the ICU liaison nurses explained their familiarity with the patient or clinical area, using situational knowledge to demonstrate heightened situational awareness to support their MET call stand‐down decision‐making.

Reported exemplars of the influence of situational knowledge included:

…no bloods were done from the previous MET call and that was supposed to happen, and IV (intravenous) fluids were also supposed to be given …and that didn't happen either…, so they [the bedside nurses] needed to chase all that up now before I left. [Interview, N1]

N1 outlines how they use the previous MET call to identify gaps in the patient's management plan that need to be addressed before ending the MET call.

ICU liaison nurses were observed and reported to be influenced by propositional, experiential, and situational knowledge when making the decision to stand‐down a MET call response for a patient.

#### 5.3.4 Information Processing Styles

ICU liaison nurses in this study were observed to use different information processing styles and within this decision‐making shortcuts known as heuristics to support them in making a quick and efficient decision to end a MET call. This heuristic‐driven behaviour at times presented as clinician bias. There was evidence of individualised assessments of patient situations and decision‐making behaviour influenced by heuristics. Examples of heuristics were spread across two types of knowledge, that is, experiential and situational. Heuristics used by the ICU liaison nurses included: affect heuristic, availability heuristic, familiarity heuristic, and the representative heuristic.

The affect heuristic involves making decisions based on emotions or feelings associated with a situation rather than objective analysis (Chen et al. 1996; De Neys et al. 2005; Evans 2003). It is a cognitive shortcut that enables decision‐makers to quickly and efficiently solve problems where emotional responses and non‐verbal cues influence their decisions. ICU liaison nurses might decide to stand‐down a MET call if they have a positive emotional response to the patient's current condition or if the situation seems less urgent based on their emotional assessment. For example, if the patient appears stable and not in distress, the nurses might feel more confident in standing down the call.

N1 stated ‘…. [the patients] look at you and you can tell they're not okay or that they are…it's based on the fact that I see these types of patients [neuro] quite a lot. There are nonverbal cues as well as their (the patient's) response to treatments sometimes…’ [Interview, N1]

The availability heuristic is a cognitive shortcut used by decision‐makers, relying on information that is easily recalled when making a decision (Chen et al. 1996; De Neys et al. 2005; Evans 2003). ICU liaison nurses may base their decision to stand‐down a MET call on recent similar cases, where standing down was appropriate, or if they recall recent situations where the patient did not require additional intervention after a MET call. If they have encountered many cases where, standing down was not problematic, they might be more inclined to use this heuristic.

Some observed and reported examples of this included:

She [N1] is discussing the blood gas result with the ICU clinical lead [senior doctor] on the phone and has stated: ‘his [the patient's] arterial oxygen level is very low, even though he is on 15 litres via the non‐rebreather mask’ [N1]. She [N1] is talking to the ICU clinical lead [senior doctor] about non‐invasive ventilation (NIV) requirements for the patient and the treating team registrar [senior doctor] has asked if the patient can go to the Intensive Care Unit (ICU). She has replied: ‘maybe’ [N1]. N1 is reiterating to the treating team resident [junior doctor] and to the nurse‐in‐charge that the patient's blood gas result is terrible and that they [treating team resident] must contact the NIV team. [Observation N1]

… his [the patient's] airway, breathing, circulation was all safe […]they [the doctors] were getting a plan to check his haemoglobin (Hb) because his [the patient's] blood pressure was low, so I was happy about that [Interview with N5]

The familiarity heuristic involves decision‐making being influenced by interpreting current situations as similar to previous ones (Chen et al. 1996; De Neys et al. 2005; Evans 2003). As a result, decision‐makers may choose or judge based on their past decisions, especially when dealing with a high cognitive load. The ICU liaison nurses in this study were observed and reported to use this mental shortcut when using experiential and situational knowledge to support them in determining whether or not to end a MET call.

Some examples of this included:

I've had a lot of experience with these types of patients [neuro patients] in ICU (Intensive Care Unit) so I know when to end the MET call based on that. [Interview with N1]

I see a lot of these patients in MET calls […], his [the patient] oxygen saturations were still very low, so we put him [the patient] back on oxygen and then I waited till his oxygen saturations were normal and then I left…. [Interview with N2]

N2 is explaining that it is a really common issue for patients in this area to *continue to deteriorate requiring another MET call*. N2 states: ‘*they have tendency to fall under the radar of the treating medical team and that in their experience they often get missed’* [N2] [Observation N2]

The representative heuristic is applied when a decision‐maker evaluates and categorises items based on their resemblance to each other, especially in situations of uncertainty. ICU liaison nurses were observed to use this heuristic, drawing on experiential and situational knowledge to inform their decision to end the MET call.

Some representative examples of this include:

She [N7] is letting the treating team resident [junior doctor] know that the patient has slightly unequal pupils and that he [the patient] is complaining of a frontal headache and has a slightly sluggish right pupil specifically. She states: *‘in my previous experience this could mean something serious is happening neurologically’ [N7]* [Observation N7]

N5 is discussing with the medical registrar [senior doctor] and treating team registrar [senior doctor], if they are sure they want to give amiodarone, given that in all the previous MET calls [for this patient] and in this MET call, the patient has been both tachycardic and bradycardic and the potential risks with giving amiodarone. [Observation N5]

## 6 Discussion

To our knowledge, this is one of the first studies that has sought to investigate the intrinsic influences that impact ICU liaison nurses' decision to stand‐down a MET call. Our research uncovered several types of intrinsic influence that play a crucial role in the stand‐down decision‐making process. These influences revolve around ICU liaison nurses using three distinct types of knowledge: propositional, experiential, and situational. In addition, to expedite decision‐making, there was evidence of ICU liaison nurses using different information processing styles, which included evidence of biases and heuristics. ICU liaison nurses used these types of knowledge and approaches, together with environmental information, to determine whether it was appropriate to end a MET call response and safely transition the patient into the post‐MET call phase. Using an inductive and explorative approach allowed researchers to capture the decision‐maker's perspective on the various influences that impact their decision to end a MET call.

We found that the types of decisions were influenced by intrinsic variables that are similar to influences shown in other research of nurses' decision‐making under pressure (Nibbelink and Brewer 2018). This paper adds to the evidence on types of knowledge influencing decision‐making and provides new evidence of real‐world decision‐making on the information processing styles (heuristics and biases) that impact nurses. Literature has demonstrated that the cognitive and implicit biases of healthcare practitioners are a patient safety concern and lead to adverse events (Croskerry 2002, 2009; Thirsk et al. 2022).

The ICU liaison nurses used propositional knowledge to support their decision to end a MET call response. They gathered and verified empirical information from multiple sources, including patient assessment data, the electronic medical record (eMR), hospital protocols and policies, and devices/monitoring to support their decisions to end a MET call. This is consistent with early seminal studies that have identified that nurses obtain this propositional knowledge via their education, integrating research evidence at the bedside to make complex and high‐stakes decisions (Cioffi 2000; Corcoran 1986; Hamm 1988). Previous salient research also emphasises that propositional knowledge is essential for critical care nurses to identify crucial patient changes to make clinical decisions, encompassing research, theory, and information (Bucknall et al. 2007), which needs to be observable, factual, descriptive, replicable, and generalisable (Hutchinson and Bucknall 2016; Kuhn 2012). Reflective of the literature, ICU liaison nurses in our study used this propositional knowledge to support their assessment of key patient changes and, therefore, patient stability or status. This also assisted them in making decisions regarding the patient's management plan and the subsequent MET call stand‐down.

If propositional knowledge on its own did not satisfy the ICU liaison nurse in deciding to end a MET call, then they used their experiential knowledge to support their decision‐making. Interestingly, this use of experiential knowledge was further evident in the more experienced nurses (> 16 years). This finding is anticipated, as the ICU liaison nurse's expertise is built through encountering similar patients, MET diagnoses, MET call trajectories, ward settings, staff, and equipment. These experiences helped them recognise their familiarity or lack of familiarity with a specific MET call event. They used this to determine when to stand‐down a MET call response by comparing the current situation with past patterns and similar cases. This reflects several sentinel studies that also identify that experiential knowledge is gained from clinical experience and repeated exposure to similar types of clinical areas, patients, and patient treatment responses (Benner and Tanner 1987; Corcoran 1986; Hamm 1988). Another primary study reiterates that nurses gain experiential knowledge through hands‐on clinical experience, helping them better understand the ‘how’ and ‘why’ of clinical situations, to improve their perception of cues, and enhance their ability to prioritise tasks (Bucknall et al. 2007). The ICU liaison nurses in our study mentioned their familiarity with certain patients or clinical areas and the staff within those areas, drawing on their experience to determine their decision to end a MET call.

The identification of similarities between patient circumstances is an awareness and skill that develops temporally (Benner and Tanner 1987). Other primary studies have shown that expert nurses, such as ICU liaison nurses, use their experience to improve their decision‐making and rely on recognising familiar clinical situations to increase their confidence in their decision‐making (Benner and Tanner 1987; Corcoran 1986). Nibbelink and Brenner (2019) in their recent study suggest that recognising patterns can be seen as using ‘nursing intuition’ and that familiar characteristics of a situation can influence decision‐making. This study demonstrated that the ICU liaison nurse's decision to stand‐down a MET call response was influenced by identifying deterioration patterns, expected treatment responses, and predicting the MET call trajectory based on prior experiences and expertise.

The ICU liaison nurses used situational knowledge to support their MET call stand‐down decisions. This influenced their comprehension of a specific patient situation; MET call diagnosis, ward area, and staff group. Reflective of other studies, situational knowledge use in our study focused on the ICU liaison nurse's familiarity with the clinical environment, staff, patient situation, and treatment protocols (Nibbelink and Reed 2019; Stubbings et al. 2012). Similarly, Bucknall et al. (2007) identify that situational knowledge also results from repeated exposures to specific clinical situations and familiar settings. Much like experiential knowledge, it influences a nurse's decision‐making through continuous comparison of data with prior experiences, including individual patients, their caregivers, staff, equipment, policies, and procedures (Bucknall et al. 2007). The ICU liaison nurses in our study were also observed to confirm, compare, and collect more information to support their decision to end a MET call.

Subsequently, the ICU liaison nurses would expand their patient assessment (Nibbelink and Reed 2019), reiterate essential information, and confirm evidence using their situational knowledge to improve their familiarity with the MET call environment, people (staff within the MET call and the patient situation), and protocols (Braaten 2015; Pantazopoulos et al. 2012; Stubbings et al. 2012). Two key studies supporting this finding outline that understanding the current patient state or status is viewed by nurses as an essential component to increasing situational awareness and that this situational knowledge informs their subsequent decision‐making practice (Stubbings et al. 2012; Zsambok 1997). Understanding patient status is linked to recognising key patient cues (Braaten 2015; Pantazopoulos et al. 2012) and interpreting the information to better understand potential outcomes of a decision made (Stubbings et al. 2012; Zsambok 1997).

Nurses like the ICU liaison nurses in our study often make complex decisions in their practice, and various theories have been proposed to explain their decision‐making processes (Nibbelink and Brewer 2018). Dual‐process Decision‐Making Theory hypothesises that decisions are made through two systems: a fast, intuitive, and automatic system and a slower, analytical, and deliberate system (Croskerry 2009). ICU liaison nurses in our study used different information processing styles and specifically the automatic system (fast and intuitive), where they were often observed to use heuristics or ‘shortcuts’ to accelerate and simplify their decision‐making. One salient study has identified that these heuristics create biases that become the discrepancy between desired behaviour and the behaviour influenced by these shortcuts (Gilovich et al. 2002). Several other studies have demonstrated that these cognitive and implicit biases may contribute to adverse events and pose a major problem with respect to clinical decision‐making, affecting clinician interactions, treatment decisions, and patient outcomes (Croskerry 2009, 2013; Croskerry et al. 2013b; Thirsk et al. 2022) The ICU liaison nurse participants' decision‐making behaviour demonstrated the use of affect, availability, familiarity, and representative heuristics (Gilovich et al. 2002).

The ICU liaison nurses were observed to use the affect heuristic to support their decision to stand‐down a MET call response. ICU liaison nurses used their experiential and situational knowledge to rapidly assess patient status and the ward‐based clinicians' comfort level in resuming patient care. This is reflective of the literature which describes the affect heuristic as a mental shortcut that allows decision‐makers to quickly and efficiently solve problems, using their perception of others emotions to support their decision‐making (Chen et al. 1996; De Neys et al. 2005; Evans 2003).

The ICU liaison nurses also relied on the availability heuristic to make fast MET call stand‐down decisions. They verbalised key pieces of recalled information concerning the patient's condition to support their decision to end the MET call. This finding is also found in the literature that describes the availability heuristic as a mental shortcut that relies on the decision‐maker recalling information from their short‐term or working memory when deciding under time constraints (Chen et al. 1996; De Neys et al. 2005; Evans 2003).

The ICU liaison nurses used the familiarity heuristic when they had prior knowledge of a MET call situation, diagnosis, clinical area, or experience with a similar MET call trajectory. They would often make decisions about ending a MET call based on what they had previously done if the outcome was positive that is, the patient did not experience further deterioration. Decision‐making literature explains that the familiarity heuristic is based on the availability heuristic (De Neys et al. 2005). This literature supports that decision‐making can be influenced by interpreting current situations as similar to previous ones, especially when under high cognitive load, which often occurs in a MET call situation (Chen et al. 1996; De Neys et al. 2005; Evans 2003). The ICU liaison nurses were frequently observed to receive concurrent calls for MET review. This placed them under duress to make quick and efficient decisions whilst making sure patient safety was paramount.

Finally, the ICU liaison nurses used the representative heuristic when dealing with unfamiliar MET situations to compare information and evidence to support their decision to end a MET call. This is consistent with prior studies showing that decision‐makers use the representative heuristic to make decisions or judgements when faced with uncertainty, basing their choices on similarity and expecting causes and effects to be alike (Chen et al. 1996; De Neys et al. 2005; Evans 2003).

If adverse events are systematic and predictable, then there is an opportunity to develop standardised approaches to the decision to end a MET call to reduce bias and improve decision‐making (Thirsk et al. 2022). Dual‐process decision‐making theory recognises that nurses' decision‐making processes are influenced by a range of factors, including knowledge and experience and situational factors (Nibbelink and Brewer 2018). By understanding these influences, ICU liaison nurses can reflect on the types of knowledge used and their information processing styles, inclusive of cognitive biases and the use of heuristics, to improve their decision‐making processes and support undertaking safer and more effective MET call stand‐down decisions. In identifying these information processing styles, then strategies for debiasing decisions in practice can be developed and tested (Croskerry et al. 2013b, 2013a).

## 7 Limitations

The study design and sampling technique may impede the generalisation of findings beyond the study population. It should also be noted that there was uneven distribution of MET call patient attendances across the ICU liaison nurse group due to COVID staffing shortfalls and sick leave; the sample size was small, including only seven ICU liaison nurses at a single study site, and there was also a limited timeframe for interviews post‐MET call. To counter this, high numbers of MET call responses were observed.

Notably, the Hawthorne effect, where participants change or improve their behaviour because they are being observed (Adair 1984; Sedgwick and Greenwood 2015) and the Rosenthal phenomenon, where the outcome of a study is influenced by the observer's expectations (Grim 1984), could have impacted the results due to the non‐participant observer's prior professional relationship with the ICU liaison nurse group. However, to overcome this, the observer explained their non‐participant observer role and conducted post‐MET call interviews with the participants to ensure triangulation of observational data and to reduce bias. Transcripts were reviewed by the research team, a reflexive diary was kept by the observer, and assumptions and roles were discussed in meetings. It should also be noted that this study did not include patient outcomes or adverse incidents related to the MET calls.

## 8 Conclusion

Optimising nurse decision‐making is fundamental to reducing risk and promoting patient safety. This study described the intrinsic influences that impact individual ICU liaison nurse decision‐making and their decision to end a MET call. By understanding decision‐making processes and the influences on decision‐making, this study provides new insight into a critical patient safety intervention and an opportunity for developing strategies to enhance decisions and prevent biases that may lead to decision‐making errors.

## Author Contributions

**Natalie A. Kondos:** conceptualisation, investigation, methodology, data curation, writing – original draft preparation, reviewing, editing, and project administration. **Tracey Bucknall:** conceptualisation, methodology, formal analysis, supervision, writing – review and editing. **Jo McDonall:** methodology, supervision, writing – review and editing. **Jonathan Barrett:** supervision, writing – review and editing. We have no known conflict of interest to disclose and no funding to disclose.

## Conflicts of Interest

The authors declare no conflicts of interest.

## Supporting information


**Data S1:** jan70148‐sup‐0001‐DataS1.docx.

## Data Availability

Research data are not shared.
