# Supplementary material for: Intrinsic Influences on Medical Emergency Team Call Stand‐Down Decision‐Making: An Observational Study
Source: J Adv Nurs. 2025 Sep 16;82(5):5148–61. doi: 10.1111/jan.70148 (PMC13069203; doi:10.1111/jan.70148)
Supplement: Supplementary file 1 — Data S1: jan70148‐sup‐0001‐DataS1.docx. [file JAN-82-5148-s001.docx]

**Supplementary file 1**

**32-item COREQ checklist:**

| **Domain 1: Research team and reflexivity** | **Response** | **Page number reference** |
| --- | --- | --- |
| **Personal Characteristics** |  |  |
| 1. Interviewer/facilitator Which author/s conducted the interview or focus group? | Student researcher | Pg. 9-10  Pg 12-13 |
| 2. Credentials What were the researcher’s credentials? E.g. PhD, MD | PhD candidate | Pg. 12-13 |
| 3. Occupation What was their occupation at the time of the study? | Resuscitation Nurse Educator | Pg. 12-13 |
| 4. Gender Was the researcher male or female? | Female | Pg. 12-13 |
| 5. Experience and training What experience or training did the researcher have? | received training in data collection and qualitative methods as part of PhD candidature | Pg. 12 |
| **Relationship with participants** |  |  |
| 6. Relationship established Was a relationship established prior to study commencement? | Yes-in a non-clinical professional capacity | Pg. 12-13 |
| 7. Participant knowledge of the interviewer  What did the participants know about the researcher? e.g. personal goals, reasons for doing the research | The participants were aware of the researcher's purpose and aim for the research study, which focused on practice improvement. | Pg. 12 |
| 8. Interviewer characteristics What characteristics were reported about the interviewer/facilitator? e.g. Bias, assumptions, reasons and interests in the research topic | Before the study began, the interviewer characteristics, including potential biases and risks to participants, were disclosed via an introductory email and PLS (Appendix 1). | Pg. 12 |
| **Domain 2: study design** |  |  |
| **Theoretical framework** |  |  |
| 9. Methodological orientation and Theory:  What methodological orientation was stated to underpin the study? e.g. grounded theory, discourse analysis, ethnography, phenomenology, content analysis | Inductive Content Analysis was used | Pg. 10-11 |
| **Participant selection** |  |  |
| 10. Sampling How were participants selected? e.g. purposive, convenience, consecutive, snowball | Purposive sampling was used for the MET call observation as ‘real-time’ notification of MET calls is relayed via the hospital’s overhead speaker alert system, with this communication of deteriorating patient escalation already in place. Convenience sampling was used to conduct separate post MET call interviews to ensure that the ICU liaison nurses involved in the MET call could describe their own decision-making practice relative to the MET call previously under observation. | Pg. 9 |
| 11. Method of approach How were participants approached? e.g. face-to-face, telephone, mail, email | A third party was used to obtain general consent from participants in the study via email to reduce the influence of the student researcher. The ICU medical and nursing clinical leads for the hospital recruited participants to minimise this risk of coercion.  All seven potential participants were notified via email about the purpose of the study and invited to participate. The email contained a brief study overview, a Plain Language Statement (PLS) (Appendix 1) which included the aims of study and what participating in the study would require, and a withdrawal of consent form (Appendix 2). Hard copies of this PLS (Appendix 1)and withdrawal of consent form (Appendix 2) were also given to those recruited at the beginning of MET call observation session for a given day. | Pg. 10  Pg. 7-8 |
| 12. Sample size How many participants were in the study? | Seven ICU liaison nurses participated in the study |  |
| 13. Non-participation How many people refused to participate or dropped out? Reasons? | None- all of those recruited participated | Pg. 7-8 |
| **Setting** |  |  |
| 14. Setting of data collection Where was the data collected? e.g. home, clinic, workplace | The study took place at a single site large acute tertiary referral public hospital, with a mature three-tiered rapid response system. The hospital has 650 acute admission beds and approximately 9,000 MET calls are escalated annually. To ensure COVID-19 safe principles and to minimise potential transmission risk for COVID-19, COVID acute clinical areas and patients who were under airborne precaution isolation were excluded in this study. | Pg. 8 |
| 15. Presence of non-participants Was anyone else present besides the participants and researchers? | A waiver of informed consent was provided for the MET call patients and all other clinicians involved in the MET call- this included the MET call patient, bedside nursing staff, local treating medical team, pharmacists and allied health staff, nursing and medical staff and any family members or carers present | Pg. 8 |
| 16. Description of sample What are the important characteristics of the sample? e.g. demographic data, date | See Table 1 | Pg. 14-15 |
| **Data collection** |  |  |
| 17. Interview guide Were questions, prompts, guides provided by the authors? Was it pilot tested? | See Appendix 1- yes it was piloted tested and interrater reliability testing was performed on 5 of the observed MET calls to ensure that it generated consistent responses | Pg. 9  Pg. 10  Appendix 1 |
| 18. Repeat interviews Were repeat interviews carried out? If yes, how many? | No repeat interviews were carried out- phone call interruptions were managed within the interview time | Pg. 9 |
| 19. Audio/visual recording Did the research use audio or visual recording to collect the data? | Yes- audio recording was used for the observations and interviews | Pg. 9-10 |
| 20. Field notes Were field notes made during and/or after the interview or focus group? | Yes-field notes were kept for both the observations and interviews | Pg. 10 |
| 21. Duration What was the duration of the interviews or focus group? | Up to 8-15 minutes for interviews | Pg. 9 and Pg. 14 |
| 22. Data saturation Was data saturation discussed? | Yes- it was discussed before the data collection that 50 MET calls would suffice to capture the seven participants in the observation and interviews- the time period for collection was extended to due staffing challenges and sick leave secondary to COVID-19 | Pg. 10  Pg. 14 |
| 23. Transcripts returned Were transcripts returned to participants for comment and/or correction? | No transcripts were not returned to participants however post-observation interviews were conducted to support participant validation and confirmation of observational data and findings | Pg. 10 |
| **Domain 3: analysis and findings** |  |  |
| **Data analysis** |  |  |
| 24. Number of data coders How many data coders coded the data? | Three researchers supported coding the data | TBC |
| 25. Description of the coding tree Did authors provide a description of the coding tree? | No | n/a |
| 26. Derivation of themes Were themes identified in advance or derived from the data? | No this was inductive content analysis approach, so theme/sub-themes were generated from the data | Pg. 10-11 |
| 27. Software What software, if applicable, was used to manage the data? | - NVivo 11 Pro software aided data management, coding, and categorization  - Microsoft Excel (version 360) and SPSS software (version 27.0) were used for demographic data analysis | Pg. 11  Pg. 11 |
| 28. Participant checking Did participants provide feedback on the findings? | -No as previously discussed post-observation interviews were used to validate/confirm observational data collected | Pg. 10 |
| **Reporting** |  |  |
| 29. Quotations presented Were participant quotations presented to illustrate the themes / findings? Was each quotation identified? e.g. participant number | Yes quotations from each participant were used and these participants were given a unique case number i.e. N1-N7 | Pg. 23-27 |
| 30. Data and findings consistent Was there consistency between the data presented and the findings? | Yes | Pg. 14 - 27 |
| 31. Clarity of major themes Were major themes clearly presented in the findings? | Yes- please see table 5- intrinsic influences on ICU liaison nurse’s decision-making practice | Pg. 23-27 |
| 32. Clarity of minor themes Is there a description of diverse cases or discussion of minor themes? | Yes- please see tables 1-4, provides demographic data for participant group and MET call location, characteristics i.e., duration and concurrent MET calls | Pg. 14-22 |
